# The Royal Free Hospital

**Published:** 1907-11-23

**Authors:** 

**Affiliations:** President.; Chairman of the Committee.


### THE ROYAL FREE HOSPITAL.

*To the Editor of* The Hospital.

Sir,—We ask your help in making known the special needs of the Royal Free Hospital.

The weekly cost of the work there is about £300. During the present year the Committee have been obliged to build and equip two new operating-theatres, and to carry out other structural improvements rendered needful by the greatly increased work of the hospital. These improvements have been strongly recommended by the Visitors of King Edward's Hospital Fund, and are now nearly finished. They have cost about £10,000, and, unless this amount is raised forthwith, it may be found necessary to reduce the work of the hospital. The hospital stands in the Gray's Inn Road, in a poor and crowded district. Being near to several large factories and railway-stations, it has constantly to deal with most severe and urgent surgical cases. Treatment is given each year to about 2,500 in-patients and 40,000 out-patients, including casualties and maternity cases. Since its foundation in 1828 nearly three million persons have benefited by the hospital. Help is never refused in any urgent case, but, to prevent any misuse of the charity, inquiry is always made as to the means of applicants.

As indicated by the title, admission to the hospital is entirely free. No subscribers' letters are required, and no charge is made to the patients. The ordinary annual expenditure is about £16,000, while the assured income does not exceed one-third of that sum.

Contributions will be thankfully received by Lloyds Bank Limited, at any of its branches, and by the Treasurers and the Secretary at the hospital.

Yours faithfully,

Helena, President.

Sandwich, Chairman of the Committee.

